# Effect of an enamel matrix derivative (Emdogain) on the microhardness and chemical composition of human root dentin: an in vitro study

**DOI:** 10.1038/s41598-022-13081-9

**Published:** 2022-05-25

**Authors:** Karime Tavares Lima da Silva, Renata Grazziotin-Soares, Rafael Resende de Miranda, Veridiana Resende Novais, Edilausson Moreno Carvalho, Gisele Rodrigues da Silva, Jose Bauer, Ceci Nunes Carvalho

**Affiliations:** 1 Postgraduate Program of Dentistry, CEUMA University, São Luís, Maranhão Brazil; 2 Department of Dentistry, Instituto Florence, São Luís, Maranhão Brazil; 3 Department of Operative Dentistry and Dental Materials, School of Dentistry, Federal University of Uberlândia, Uberlândia, Minas Gerais Brazil; 4 College of Dentistry, University of Saskatchewan, Saskatoon, SK Canada; 5 Dentistry Biomaterials Laboratory (Biomma), School of Dentistry, University Federal of Maranhão (UFMA), São Luis, MA Brazil

**Keywords:** Materials science, Biomaterials, Biomedical materials

## Abstract

The advantage of using an Enamel matrix derivative EMD Emdogain as an intracanal medication could be a manner to strength the tooth structure, improving the physical and chemical properties of dentin. We tested, in vitro, the effect of Emdogain on the surface microhardness and chemical composition of root dentin. Ten human teeth were used to produce dentin specimens originated from the canal walls (n = 30) that remained in contact to Emdogain gel for 90 days. Baseline and 90-days after Emdogain treatment measurements were performed using Fourier Transform Infrared Spectroscopy (ATR/FTIR), Scanning Electron Microscopy/Energy Dispersive Spectroscopy (SEM/EDS) and Knoop indenters. The use of EMD (Emdogain) for 90 days in contact with human root canal dentin specimens did not alter the microhardness and morphology of dentin. The elemental structure of dentin was altered because there was a reduction in carbonate content.

## Introduction

Around 1981 scientists found that certain enamel matrix derivatives (EMDs)—which until then were considered tooth enamel specific proteins—played a role in the cementum formation when they were deposited on the surface of developing tooth roots^1^. These observations led to the hypothesis that EMPs could play an integral role in the differentiation of periodontal tissues and have been the basis of several biological and clinical studies. Later, it was demonstrated that, during the development of the human dental-pulp complex, the secretion of endogenous enamel matrix derivative (EMD) by the Hertwig’s epithelial root sheath triggered a cascade of reactions that stimulate odontogenesis^2,3^. In addition to that, the Hertwig’s sheath deposited enamel matrix proteins on the root surface prior to cementum formation, and these proteins were the initiating factor for cementogenesis^4^.

Emdogain is the commercial name for a synthetic gel containing EMD. EMD is a protein extract from unerupted porcine tooth buds that contains approximately 90% amelogenins and smaller amounts of tuftelin, ameloblastin, enamelin, and other nonamelogenin proteins^5^. The mixture of these natural proteins may induce biological processes that usually take place during the development of the periodontium and to stimulate cells involved in the healing process of soft and hard tissues. Clinically, the Emdogain has been traditionally used for the treatment of periodontal bony defects and soft tissue recession^6–8^.

In endodontics, the EMD (Emdogain) has been mostly studied as an adjunct to improve regenerative approaches^5,9,10^. The potential of EMD in regenerative endodontics is not yet fully understood, but it is proved that EMD has an important role in odontogenesis, improving pulp tissue healing and regeneration^11^. When the EMD is used as an adjunct to a conservative pulp treatment (direct pulp capping or pulpotomy, for example), it induces the formation of reparative dentin, protecting the pulp tissue and consequently preventing pulp degeneration^12–15^. The endodontic literature has reported potential advantages of using Emdogain in surgical sealing of root perforations^16^, direct pulp capping^17,18^ pulpotomy^19,20^, pulp regeneration in rats^21^, and as intracanal medication^22^. Although EMD Emdogain has been investigated as a potential substance for use in endodontics, producing good results in relation to its clinical indications, the information on its influence in dentinal properties has not been addressed.

When necrosis and apical periodontitis occur in an immature tooth, the tooth structure may become weaken, because of the incomplete apposition of dentin in the root canal walls and incomplete root development. Regenerative procedures to induce pulp revascularization is one of the options to treat these type of cases—and the use of EMD as an intracanal substance could be a manner to strength the tooth structure, improving the physical and chemical properties of dentin. Therefore, this in vitro investigation aimed to test if the EMD-gel (Emdogain) when used for 90 days had influence on mechanical or the surface chemical composition of dentin. The null hypotheses tested were: (1) EMD (Emdogain) would not alter the root dentin Knoop microhardness or morphology; and (2) EMD (Emdogain) would not affect the calcium and phosphorus content of root dentin or the ratios of mineral/matrix, carbonate/mineral, amide I/amide III and amide I/CH2.

## Material and methods

Enamel matrix derivative EMD (Emdogain) was obtained from the manufacturer (Institut Straumann AG, Basel Switzerland). For this experiment approximately 6 gel-syringes of the product were needed. Ten human teeth, extracted for therapeutic reasons, were used to produce dentin specimens. Informed consent was obtained from all participants in this study. This project was approved by the local Research Ethics Committee (approval number: CAAE 15975419.1.0000.5084).

Inclusion criteria for teeth comprised complete root formation, no radiographic signals of calcification/mineralization (nor diffuse neither localized), no evidence of internal resorption, and absence of previous endodontic initiated therapy or root canal obturation.

### Specimens’ preparation

**Figure 1:**
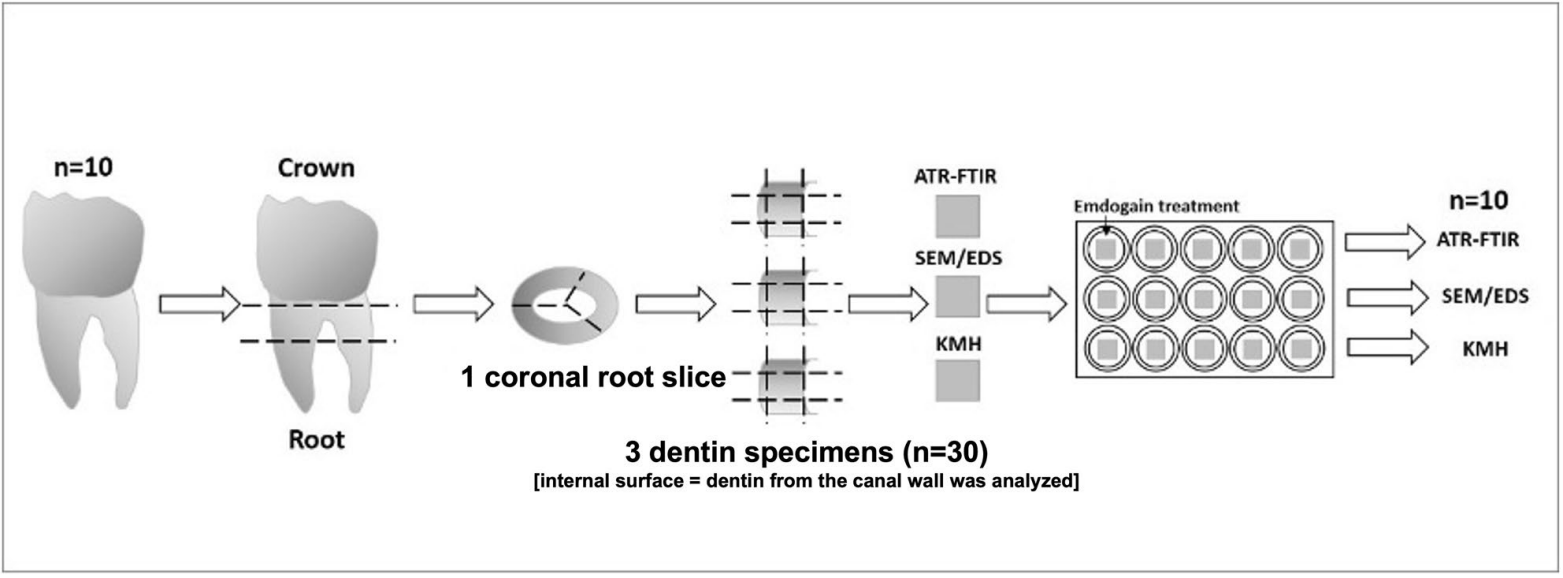
Schematic illustration of the experimental design.

Ten teeth were cleaned and maintained in distilled water for a maximum of 6 months after extraction. Firstly, the crown was separated from the roots using a diamond disc attached to a cutting machine (Isomet 1000 Precision Saw Buehler). The tooth crown was discarded. Secondly, one cross-sectional slice was acquired from each tooth root (from the coronal region of the root). The cross-sectional slice was, then, split (longitudinally) to produce 3 squared specimens—where the internal surface of each specimen (dentin from the canal walls) was analysed. This means that, 10 teeth originated 3 dentin specimens (n = 30). Specimens measured 3 mm diameter × 3 mm height (Fig. 1).

For SEM/EDS analysis and microhardness test, the specimens were built-in synthetic plastic polymer (polyvinyl chloride tubes) using acrylic resin (TDV, Pomerode, SC, Brasil) for fixation of the disk base. The disk dentin surfaces were polished using silicon carbide sandpaper in decreasing grains (#400, #600 and #1200). The internal surface of each dentin (the surface to be analyzed) specimen was polished with felt cloths soaked in diamond paste attached to a slow-speed handpiece (Diamond, FGM, Joinville, SC, Brazil). Then, specimens were washed in an ultrasonic vat with distilled water for 30 min. After taking the baseline measures, the specimens were stored in an oven at 37 °C, under humidity (dentin specimens remained in well plates covered with 2 × 2 gauze moistened with distilled water).

### Enamel matrix derivative (EMD) (Emdogain) treatment

EMD (Emdogain) was injected directly from the manufacturer syringes into petri dishes. The dentin specimens were then set down over the EMD, having one of the disk surfaces (the surface to be analyzed) immersed into the substance. Specimens remained for 90 days into an incubator at 37 °C and 100% humidity. After EMD treatment, the specimens were again washed in an ultrasonic vat with distilled water for 30 min, and new measurements for the tests (FTIR, SEM/EDS and MH) were obtained.

### Chemical composition with fourier transform infrared spectroscopy (FTIR)

Chemical composition of the samples (n = 10) was determined using attenuated total reflectance/Fourier transform infrared spectroscopy (ATR/FTIR; Vertex 70, Bruker, Ettlingen, Germany). Evaluations were made before and 90-days after EMD (Emdogain) treatment. The dentin surfaces (without being included in acrylic blocks) were positioned against the diamond crystal of the ATR/FTIR unit. Spectra were recorded in the range from 400 to 4000 cm^−1^ at 4 cm^−1^ of resolution. Each specimen was scanned 32 times in each FTIR measurement, and the final spectrum acquired was the average of all these scans. Spectra were recorded and analyzed by OPUS 6.5 software (Bruker, Ettlingen, Germany). After baseline correction and normalization, the area under each band was integrated by using the appropriate tools from the software. Each spectrum was normalized according to the phosphate band (1190–702 cm^−1^). FTIR spectra were analyzed by calculating the following parameters: (1) mineral/matrix ratio M:M (the ratio of the integrated areas of phosphate v1, v3 stretching mode at 1,035 cm^−1^ to the collagen amide I at 1.655 cm^−1^); (2) carbonate/mineral ratio C:M (the ratio of the integrated areas of carbonate v2 at 872 cm^−1^ to the phosphate v1, v3 at 1,035 cm^−1)^; (3) amide I/amide III ratio (the ratio of the integrated areas of amide I at 1655 cm^−1^ to the amide III at 1235 cm^−1^); (4) amide I/CH_2_ ratio (the band ratio of the integrated areas of amide I at 1,655 cm^−1^ to the CH_2_ scissoring at 1450 cm^−1^)^23,24^.

### Surface morphology and element analysis with scanning electron microscopy/energy dispersive spectroscopy (SEM/EDS)

Images and spectra of the dentinal surfaces were obtained before and 90-days after the treatment with EMD (Emdogain) on a tabletop Scanning Electron Microscope (SEM) (TM3030, Hitachi, Tokyo, Japan). Prior to the analysis, specimens were ultrasonically cleaned and fixed in a metallic stub using a double-sided carbon tape. Samples were evaluated at 2500 × magnification in backscattered electron mode, having as reference point the central region of the sample. Subsequently, the EDS spectra were collected from the same dentinal surfaces to identify calcium and phosphorus elements, similarly to SEM images.

### Knoop microhardness (KMH)

Baseline microhardness readings were obtained from the specimen surface before the treatment with EMD (Emdogain). Dentin microhardness was measured with a Knoop indenter at 40 × magnification (Shimadzu HMV-2000; Shimadzu Corporation, Kyoto, Japan), with a load of 10 g for 15 s. The average length of the two diagonals produced by the indenter was used to calculate the KMH value. Four indentations were made in each specimen at 20 µm far away from the root canal lumen. The representative microhardness value for each sample was the average result of the four indentations. Ninety days after EMD (Emdogain) treatment, the specimens were washed in an ultrasonic vat with distilled water for 30 min, and new microhardness measurements were obtained identically as described above.

### Statistical analysis

Data were tested for normal distribution (Shapiro–Wilk’s test, p > 0.05) and equality of variances (Levene’s test, p > 0.05). FTIR and KMH data were analyzed by a paired t-test, comparing before and after EMD (Emdogain) treatment. Sigma Plot statistical package (version 12.0, Systat Software, Inc., San Jose, CA, USA) was used for analysis and a p-value of lower than 0.05 was considered statistically significant. SEM/EDS findings were descriptively reported.

### Ethics statement

All methods were carried out in accordance with relevant guidelines and regulations. All experimental protocols were approved by the following institutional ethics committee: Research Ethics Committee—CEUMA Higher Education Institution (approval number: CAAE 15,975,419.1.0000.5084—Aug 28, 2019). Informed consent was obtained from all participants in this study.

## Results

### Chemical composition (FTIR)

The mean and standard deviation values for chemical parameters and ratios obtained by FTIR are shown in Table 1. After EMD (Emdogain) treatment, the samples showed a significant decrease in the values of carbonate (p = 0.001) and amide III (p = 0.002). The C:M ratios decreased in the samples after EMD (Emdogain) treatment (p < 0.001), while the amide I/amide III increased.

**Table 1:** Mean and standard deviation (SD) of Knoop Dentin Microhardness (KMH); and mean and SD of the integrated area of each chemical component, as well as ratios (M:M; C:M, amide I/amide III and amide I/CH_2_) analyzed by FTIR before and 90-days after EMD (Emdogain) treatment.

	Before EDM treatment (baseline)	After EDM treatment	p-value
Dentin microhardness	53.2 (10.1)	49.5 (12.1)	= 0.35
**Chemical components**			
Phosphate	13.15 (1.30)	11.95 (1.79)	= 0.103
Carbonate	0.23 (0.03)	0.16 (0.03)	= 0.001*
Amide I	2.06 (0.36)	2.22 (0.73)	= 0.685
Amide III	0.22 (0.05)	0.16 (0.02)	= 0.002*
CH_2_	0.12 (0.04)	0.14 (0.05)	= 0.395
**Ratios**			
M:M	6.58 (1.40)	5.72 (1.46)	= 0.293
C:M	0.017 (0.001)	0.013 (0.001)	< 0.001**
Amide I/amide III	9.70 (1.38)	12.71 (1.71)	< 0.001**
Amide I/CH_2_	17.94 (4.43)	17.50 (6.42)	= 0.857

*Indicates differences in root dentin chemical components (in rows) obtained by paired t-test (p < 0.05).

**Indicates differences in root dentin ratios (in rows) obtained by paired t-test (p < 0.05).

**Figure 2:**
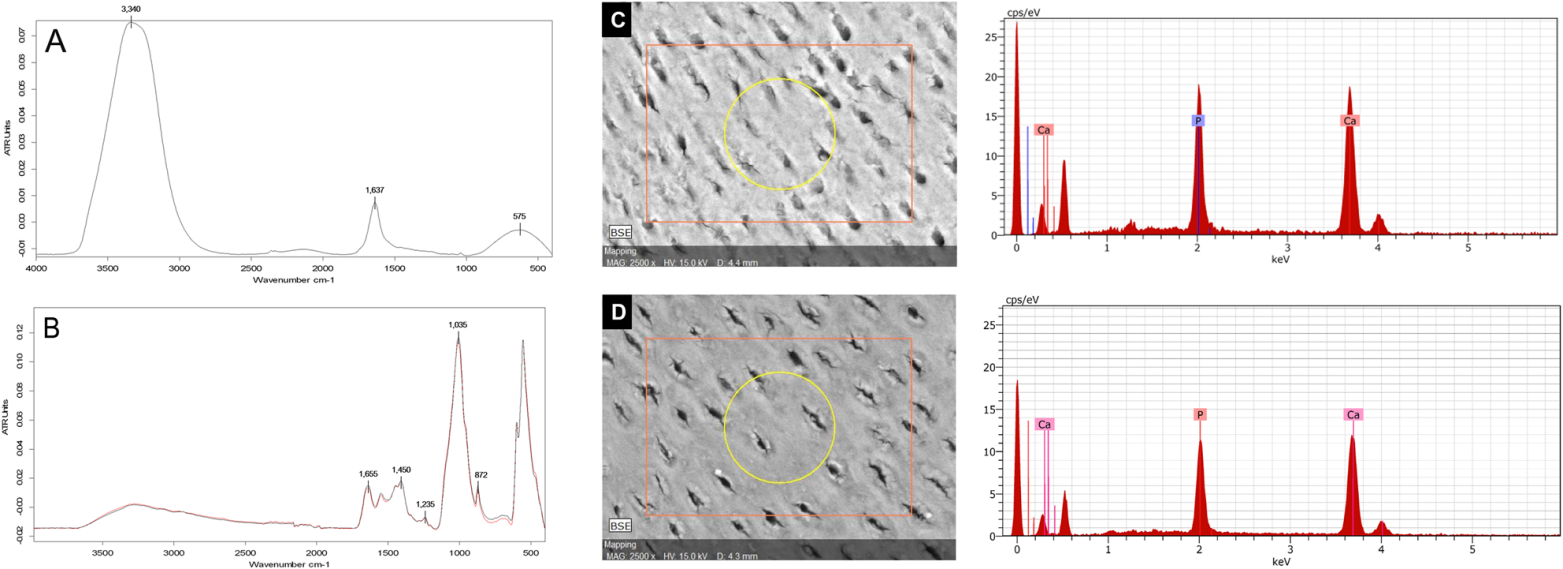
(**A**) Representative imagens of dentinal surface FTIR spectra before EMD (Emdogain) treatment. (**B**) Representative images/spectra of FTIR spectra after EMD (Emdogain) treatment. Specimens` surface from the same sample analysed by SEM/EDS: (**C**) is showing the SEM image and the spectra (right side) before treatment with EMD (Emdogain) (baseline); and (**D**) is showing the SEM image and the spectra (right side) 90-days after treatment with EMD (Emdogain).

Figure 2A shows the FTIR spectra of EMD (Emdogain). The EMD (Emdogain) spectrum presented three more evident bands observed at ∼ 575, 1,637 and 3,340 cm^-1^ wavenumbers. The peak at 3340 cm^−1^ is attributed to O–H vibrations in adsorbed water or as hydroxyl group, while the peak at 1637 cm^−1^ is dominated by the C=O stretch vibrations of the peptide linkages present in amide I^19,20^. The peak at 575 cm^-1^ is associated with phosphate v4 (PO4^3−^)^18,21^. Figure 2B shows the dentin spectra before and after EMD treatment. The peak at 1035 cm^−1^ is attributed to phosphate v1, v3 and the peak at 872 cm^−1^ to carbonate v2. The peak at 1655 cm^−1^ is associated with amide I, while the peak at 1,235 cm^−1^ is associated with amide III. The peak at 1450 cm^−1^ is attributed to the CH_2_ scissoring^17,18^.

### Surface morphology and element analysis (SEM/EDS)

Representative SEM/EDS images of specimens before (baseline) and 90-days after treatment with EMD (Emdogain) are also illustrated in Fig. 2. The SEM images showed that the EMD (Emdogain) treatment did not change the surface morphology. After 90 days, the EDS analysis showed a slight decrease in the intensity of the calcium (Ca) and phosphorus (P) peaks of the root dentin.

### Dentin microhardness (KMH)

The results of the Knoop microhardness analysis are shown Table 1. No statistically significant differences were observed in the surface microhardness of the samples before and after treatment with EMD (Emdogain) (p = 0.35).

## Discussion

The enamel matrix derivative (EMD) (Emdogain) has been indicated for promoting regeneration of dental tissues^9,10^. Emdogain is a composed mainly of amelogenin and amelin, proteins that play an important role in dentinogenesis and promote an increase in the level of mineralization markers in odontoblasts^6^. This class of proteins is known to induce the growth and proliferation of cells of the periodontal ligament, which has propylene glycol alginate (PGA) as a vehicle with an important antibacterial action^27–29^.

The literature has shown that EMD induced formation of mineralized tissue on root canal walls, by using available minerals from the dentinal tissue; this way, contributing to the root development and supporting the periapical healing^6,9,10^. In 2012, the efficacy of EMD was compared with a triple antibiotic paste (TAP) as an intracanal drug for regeneration of immature teeth in rats with pulp necrosis. EMD and TAP were able to reduce periapical lesion size loss and increase root length and thickness. EMD promoted narrower canals compared to TAP, a positive finding that could strengthen the tooth. Another experiment proved that EMD, when used in pulpotomy therapy, induced the formation of substantial amount of dentin-like tissue. The beginning of hard tissue formation was radiographically observed 2 weeks post-operative and it was located only in the affected pulp region. In comparison, the authors showed that dentin-like tissue was also formed in teeth treated with Dycal, but in limited amount, and at the expense of the whole width of the pulp chamber floor, narrowing of the root canals entrance. The total amount of dentin formed in the teeth treated with EMD was significantly higher than in the samples treated with Dycal^21^.

The findings of this present study added information to the body of knowledge on the benefits of using EMD (Emdogain) for 90 days in contact with root canal dentin. We showed that EMD (Emdogain gel) did not alter either microhardness of human dentin or its morphology, accepting the 1st null hypothesis.

The FTIR analysis is a reliable way to generate evidence about the presence of functional groups present in the structure of a sample, which can be used to identify a compound or to investigate its chemical composition^25^. Since EMD (Emdogain) is a mixture of hydrophobic enamel matrix proteins [derived from 6-month-old porcine tooth buds] containing amelogenin, enamelin, tuftelin, amelin, and ameloblastin, in a propylene glycol alginate (PGA)^30^ its proteins guide tissue regeneration and induce remineralization of enamel and dentin^31,32^.

This current study observed a reduction in dentin carbonate values after EMD (Emdogain) treatment, which also impacted the C:M ratio. This ratio indicates the extent of carbonate incorporation in the hydroxyapatite lattice^33^. As carbonate is responsible for the acidic solubility of dental hard tissues, the reduction in carbonate content is related to the increased resistance to demineralization^34^, which occurred after EMD (Emdogain) treatment. These findings rejected the 2nd null hypothesis.

Amelogenins are responsible for regulating the mineralization process and for organizing the apatite crystals into juxtaposed prisms (Moradian-Oldak, 2001). The amelogenin protein molecule is divided into three amino acid domains, which are: central domain, C-terminus (COOH) and N-terminus (NH2)^35^. Both terminus types play key roles in proteolytic processes^36^ and can interact with chemical components of the dental tissue, altering them quantitatively or changing their molecular conformation. Filamentary structure in amelogenin may induce ionic interactions, through acidic residues present in the C-terminal domain, for example^36^. This can result in modifications to the amide bands of the FTIR spectrum. In our study, these alterations found in the organic portion of dentin are represented by changes in amide III values. It is one of the amides present in the collagen structure; however, it is a very unstable and complex band depending on the details of the force field, the nature of the side chains and hydrogen bonding^24,37^. The reduction in amide III values may mean a disorganization in the secondary structure of the collagen fiber-forming protein unit^38,39^. Amide I/amide III ratio has also been altered and this represents a change in organization of collagen within the samples after Emdogain treatment^40,41^. Since collagen is the most abundant protein in dentin, its proteolysis has a significant impact on the structural integrity of this tissue, which can become mechanically and functionally compromised^23,24^. However, the chemical modifications did not repercuss in significative changes in dentin microhardness and surface morphology.

A limitation of this present study includes the assumption that the same results would be obtained in a clinical study—a challenge for any in vitro experiment. Nevertheless, we showed that EMD (Emdogain) is a potential substance to use intracanal—not interfering in dentin microhardness and contributing to increase resistance to demineralization. One of the strengthens of this study is the initial understanding of what occurs in the macro and microstructure of EMD treated dentin [the first time that it is being studied in the endodontic literature]. The findings of this current study may prompt further studies such as: tooth esthetic/color analysis when EMD is used into the root canal, analysis of other physicochemical-biological dentin properties, etc.

## Conclusion

The use of EMD (Emdogain) for 90 days in contact with human root canal dentin specimens did not alter the microhardness and morphology of dentin. The elemental structure of dentin was altered because there was a reduction in carbonate content.

## Data Availability

The datasets used and/or analysed during the current study available from the corresponding author on reasonable request.

## References

[1] Lindskog S, Hammarstrom L (1981). Formation of intermediate cementum. III: 3H-tryptophan and 3H-proline uptake into the epithelial root sheath of Hertwig in vitro. J. Craniof. Gene Dev. Biol..

[2] Sonoyama W, Seo BM, Yamaza T, Shi S (2007). Human Hertwig's epithelial root sheath cells play crucial roles in cementum formation. J. Dent. Res..

[3] Lyngstadaas SP, Wohlfahrt JC, Brookes SJ, Paine ML, Snead ML, Reseland JE (2009). Enamel matrix proteins; old molecules for new applications. Orthod. Craniofac. Res..

[4] Vishwakarma A, Shi S, Sharpe P, Ramalingam M (2015). Stem Cell Biology and Tissue Engineering in Dental Sciences.

[5] Wang HH, Sarmast ND, Shadmehr E, Angelov N, Shabahang S, Torabinejad M (2018). Application of enamel matrix derivative (emdogain) in endodontic therapy: A comprehensive literature review. J. Endod..

[6] Huang HL, Ma YH, Tu CC, Chang PC (2022). Radiographic evaluation of regeneration strategies for the treatment of advanced mandibular furcation defects: A retrospective study. Membranes (Basel)..

[7] Anoixiadou S, Parashis A, Vouros I (2022). Enamel matrix derivative as an adjunct to minimally invasive non-surgical treatment of intrabony defects: A randomized clinical trial. J. Clin. Periodontol..

[8] Windisch P, Iorio-Siciliano V, Palkovics D, Ramaglia L, Blasi A, Sculean A (2022). The role of surgical flap design (minimally invasive flap vs extended flap with papilla preservation) on the healing of intrabony defects treated with an enamel matrix derivative: A 12-month two-center randomized controlled clinical trial. Clin. Oral Investig..

[9] Sanz JL, Forner L, Almudéver A, Guerrero-Gironés J, Llena C (2020). Viability and stimulation of human stem cells from the apical papilla (hSCAPs) induced by silicate-based materials for their potential use in regenerative endodontics: A systematic review. Materials (Basel)..

[10] Karkehabadi H, Ahmadyani E, Najafi R, Khoshbin E (2022). Effect of biodentine coated with emdogain on proliferation and differentiation of human stem cells from the apical papilla. Mol. Biol. Rep..

[11] Wang Y, Zhao Y, Ge L (2014). Effects of the enamel matrix derivative on the proliferation and odontogenic differentiation of human dental pulp cells. J. Dent..

[12] Nakamura Y, Hammarstrom L, Lundberg E, Ekdahl H, Matsumoto K, Gestrelius S, Lyngstadaas SP (2001). Enamel matrix derivative promotes reparative processes in the dental pulp. Adv. Dent. Res..

[13] Nakamura Y, Hammarstrom L, Matsumoto K, Lyngstadaas SP (2002). The induction of reparative dentin by enamel proteins. Int. Endod. J..

[14] Igarashi R, Sahara T, Shimizu-Ishiura M, Sasaki T (2003). Porcine enamel matrix derivative enhances the formation of reparative dentine and dentine bridges during wound healing of amputated rat molars. J. Electron. Microsc. (Tokyo).

[15] Olsson H, Davies JR, Holst KE, Schröder U, Petersson K (2005). Dental pulp capping: Effect of Emdogain Gel on experimentally exposed human pulps. Int. Endod. J..

[16] Azim AA, Lloyd A, Huang GT (2014). Management of longstanding furcation perforation using a novel approach. J. Endod..

[17] Garrocho-Rangel A, Flores H, Silva-Herzog D, Hernandez-Sierra F, Mandeville P, Pozos-Guillen AJ (2009). Efficacy of EMD versus calcium hydroxide in direct pulp capping of primary molars: A randomized controlled clinical trial. Oral Surg Oral Med Oral Pathol Oral Radiol Endod.

[18] Orhan EO, Maden M, Senguüven B (2012). Odontoblast-like cell numbers and reparative dentine thickness after direct pulp capping with platelet-rich plasma and enamel matrix derivative: A histomorphometric evaluation. Int. Endod. J..

[19] Darwish SS, Abd El Meguid SH, Wahba NA, Mohamed AA, Chrzanowski W, Abou Neel EA (2014). Root maturation and dentin-pulp response to enamel matrix derivative in pulpotomized permanent teeth. J. Tissue Eng..

[20] Yildirim C, Basak F, Akgun OM, Polat GG, Altun C (2016). Clinical and radiographic evaluation of the effectiveness of formocresol, mineral trioxide aggregate, portland cement, and enamel matrix derivative in primary teeth pulpotomies: A two year follow-up. J. Clin. Pediatr. Dent..

[21] Scarparo RK, Dondoni L, Böttcher DE, Grecca FS, Figueiredo JA, Batista EL (2012). Apical periodontium response to enamel matrix derivative as an intracanal medication in rat immature teeth with pulp necrosis: Radiographic and histologic findings. J. Endod..

[22] Matsumoto N, Minakami M, Hatakeyama J, Haruna C, Morotomi T, Izumi T, Anan H (2014). Histologic evaluation of the effects of Emdogain gel on injured root apex in rats. J. Endod..

[23] Rodrigues RB, Soares CJ, Simamoto Junior PC, Lara VC, Arana-Chaves VE, Novais VR (2018). Influence of radiotherapy on the dentin properties and bond strength. Clin. Oral Invest..

[24] Miranda RR, Silva ACA, Dantas NO, Soares CJ, Novais VR (2019). Chemical analysis of in vivo-irradiated dentine of head and neck cancer patients by ATR-FTIR and Raman spectroscopy. Clin. Oral Invest..

[25] Lopes CCA, Limirio PHJO, Novais VR, Dechichi P (2018). Fourier transform infrared spectroscopy (FTIR) application chemical characterization of enamel, dentin and bone. Appl. Spectrosc. Rev..

[26] Zaharia A, Muşat V, Anghel EM, Atkinson I, Mocioiu O-C, Buşilă M, Pleşcan VG (2017). Biomimetic chitosan-hydroxyapatite hybrid biocoatings for enamel remineralization. Ceram. Int..

[27] Sculean A, Chiantella GC, Windisch P, Donos N (2000). Clinical and histologic evaluation of human intrabony defects treated with an enamel matrix protein derivative (Emdogain). Int. J. Periodontics Restorative Dent..

[28] Sculean A, Windisch P, Szendröi-Kiss D, Horváth A, Rosta P, Becker J, Gera I, Schwarz F (2008). Clinical and histologic evaluation of an enamel matrix derivative combined with a biphasic calcium phosphate for the treatment of human intrabony periodontal defects. J. Periodontol..

[29] Newman SA, Coscia SA, Jotwani R, Iacono VJ, Cutler CW (2003). Effects of enamel matrix derivative on Porphyromonas gingivalis. J. Periodontol..

[30] Sezici YL, Yetkiner E, Aykut Yetkiner A, Eden E, Attin R (2021). Comparative evaluation of fluoride varnishes, self-assembling peptide-based remineralization agent, and enamel matrix protein derivative on artificial enamel remineralization in vitro. Prog. Orthod..

[31] Grandin HM, Gemperli AC, Dard M (2012). Enamel matrix derivative: A review of cellular effects in vitro and a model of molecular arrangement and functioning. Tissue Eng. Part B Rev..

[32] Schmidlin P, Zobrist K, Attin T, Wegehaupt F (2016). In vitro re-hardening of artificial enamel caries lesions using enamel matrix proteins or self-assembling peptides. J. App. Oral Sci..

[33] Boskey AL, Mendelsohn R (2005). Infrared spectroscopic characterization of mineralized tissues. Vib. Spectrosc..

[34] Pereira DL, Freitas AZ, Bachmann L, Benetti C, Zezell DM, Ana PA (2018). Variation on molecular structure, crystallinity, and optical properties of dentin due to Nd:YAG laser and fluoride aimed at tooth erosion prevention. Int. J. Mol. Sci..

[35] Apicella A, Marascio M, Colangelo V, Soncini M, Gautieri A, Plummer C (2017). Molecular dynamics simulations of the intrinsically disordered protein amelogenin. J. Biomol. Struct..

[36] Moradian-Oldak J (2001). Amelogenins: Assembly, processing and control of crystal morphology. Matrix Biol..

[37] Xu C, Wang Y (2012). Chemical composition and structure of peritubular and intertubular human dentine revisited. Arch. Oral Biol..

[38] Bet MR, Goissis G, Lacerda CA (2001). Characterization of polyanionic collagen prepared by selective hydrolysis of asparagine and glutamine carboxyamide side chains. Biomacromol.

[39] Campi LB, Lopes FC, Soares L, de Queiroz AM, de Oliveira HF, Saquy PC, de Sousa-Neto MD (2019). Effect of radiotherapy on the chemical composition of root dentin. Head Neck.

[40] Salehi H, Terrer E, Panayotov I, Levallois B, Jacquot B, Tassery H, Cuisinier F (2013). Functional mapping of human sound and carious enamel and dentin with Raman spectroscopy. J. Biophot..

[41] Toledano M, Aguilera FS, Osorio E, Cabello I, Toledano-Osorio M, Osorio R (2015). Functional and molecular structural analysis of dentine interfaces promoted by a Zn-doped self-etching adhesive and an in vitro load cycling model. J. Mech. Behav. Biomed. Mat..

